# Review of sheep and goat pox disease: current updates on epidemiology, diagnosis, prevention and control measures in Ethiopia

**DOI:** 10.1186/s44149-021-00028-2

**Published:** 2021-11-15

**Authors:** Girma Zewdie, Getaw Derese, Belayneh Getachew, Hassen Belay, Mirtneh Akalu

**Affiliations:** 1 National Veterinary Institute, P. O. Box: 19, Bishoftu, Ethiopia; 2 Africa Union Pan African Veterinary Vaccine Center (AU-PANVAC), P. O. Box: 1746, Bishoftu, Ethiopia; 3 Department of Biotechnology, Koneru Lakshmaiah Education Foundation, Vaddeswaram, Gunture, AP 522502 India

**Keywords:** CaPV, Ethiopia, GTPV, Sheep pox, Goat pox, SPPV, Vaccine

## Abstract

Sheep pox, goat pox, and lumpy skin diseases are economically significant and contagious viral diseases of sheep, goats and cattle, respectively, caused by the genus *Capripoxvirus* (CaPV) of the family *Poxviridae*. Currently, CaPV infection of small ruminants (sheep and goats) has been distributed widely and are prevalent in Central Africa, the Middle East, Europe and Asia. This disease poses challenges to food production and distribution, affecting rural livelihoods in most African countries, including Ethiopia. Transmission occurs mainly by direct or indirect contact with infected animals. They cause high morbidity (75-100% in endemic areas) and mortality (10-85%). Additionally, the mortality rate can approach 100% in susceptible animals. Diagnosis largely relies on clinical symptoms, confirmed by laboratory testing using real-time PCR, electron microscopy, virus isolation, serology and histology. Control and eradication of sheep pox virus (SPPV), goat pox virus (GTPV), and lumpy skin disease (LSDV) depend on timely recognition of disease eruption, vector control, and movement restriction. To date, attenuated vaccines originating from KSGPV O-180 strains are effective and widely used in Ethiopia to control CaPV throughout the country. This vaccine strain is clinically safe to control CaPV in small ruminants but not in cattle which may be associated with insufficient vaccination coverage and the production of low-quality vaccines.

## Introduction

Livestock production has massive potential to achieve several of Ethiopia’s national and international assurances on poverty mitigation, food security, and improved nutrition. The economic contribution of the livestock subsector in the country is approximately 45% of the total worth of agricultural production (FAO 2019). Ethiopia is the home of the largest livestock population in Africa and has an estimated 39.89 million sheep, 50.50 million goats, and 65.35 million cattle. Thus, small ruminants (sheep and goats) are important components of the subsector and have been supporting the national economy of the country by generating hard currency from meat exports (CSA 2020). Likewise, this sector constitutes a significant portion of livestock production and is a source of cash income, meat, milk and wool for small farm holders (Haile et al. 2018).

Despite the huge livestock population, the current economic contributions of small ruminants in Ethiopia have not delivered the expected benefit to the national economy because of widely distributed infectious diseases of small ruminants such as sheep pox (SPP) and goat pox (GTP). These diseases are the main constraints that hinder the productivity of sheep and goats. Additionally, these diseases pose major economic threats globally, particularly in developing countries such as Ethiopia (Fentie et al. 2017).

SPP and GTP are typically infectious and economically significant viral diseases of sheep and goats in several parts of the world. They are manifested by widespread pox lesions across the skin and mucous membranes, constant fever, enlargement of superficial lymph nodes, pyrexia, generalized nodules (2 to 5 cm in diameter) on nonwool skin, generalized pocks, and often focal viral pneumonia (Bhanuprakash et al. 2011; Şevik et al. 2016). Therefore, the current review aims to discuss the effective control of SPP and GTP and highlight the current status of these diseases and their epidemiology, diagnosis and treatment.

## Literature review

### Etiology

Sheep pox, goat pox, and lumpy skin disease are economically important infectious diseases of sheep, goats and cattle, respectively. They belong to the genus *Capripoxvirus* (CaPV), in the subfamily *Chordopoxvirinae* and *Poxviridae* family (King et al. 2012). Sheep pox virus (SPPV) and goat pox virus (GTPV) are intimately linked to the lumpy skin disease virus (LSDV) of cattle and mainly affect sheep and goats, respectively. However, some virus isolates may cause mild to severe disease in both species; hence, these strains generally have transitional host specificity (Babiuk et al. 2008; Tuppurainen et al. 2014).

The central core of the virion contains genome and many viral proteins, while the capsid surrounds the core and two lateral bodies (Moss et al. 2006). The genome size of CaPV is relatively constant (~ 150 kbp). Additionally, the genome comprises large, brick-shaped, complex, double-stranded DNA and enveloped viruses (Biswas et al. 2020). It contributes approximately 150 putative genes, together with conserved genes engaged in virulence and host ranges, as well as conserved replicative and structural genes. Most of the genes in the central genomic region of CaPV are responsible for replicative mechanisms, while the terminal region contains genes that influence pathogenesis and host range functions (Zeng et al. 2014). A false lipid envelope surrounds the genome of CaPV and can be affected by many disinfectants and acids (Hosamani et al. 2004). The replication of CaPV occurs in the cytoplasm of infected cells rather than in the nucleus, a rare occurrence for viruses with double-stranded DNA genomes (Schramm and Locker 2005).

Because only a single serotype of CaPV exists, distinguishing among SPPV, GTPV and LSDV is challenging using serological techniques (Babiuk et al. 2008; Bowden et al. 2008) and antigenic (Babiuk et al. 2008) techniques. However, genetic sequencing and phylogenetic investigation of the *GPCR* (G-protein coupled receptor) and *RPO30* subunit genes encoding the 30 kD genes have been developed to discriminate among LSDV, GTPV and SPPV (Lamien et al. 2011). *P32*, *GPCR* and *RPO30* genes are highly conserved among *capripoxviruses* (Venkatesan et al. 2012; Mahmoud and Khafagi 2016).

Following the analysis of SPPV or GTPV, the losses of 5 open reading frames (ORFs) (Biswas et al. 2020) and deletion of 21 nucleotides in the *RPO30* gene of SPPV were reported (Rouby 2018). Additionally, the *B22R* gene portion showed deletion in the SPPV Romania strain compared with that in GTPV and LSDV (Chibssa et al. 2019). Hence, these findings revealed that GTPV is more closely linked to LSDV than SPPV (Le Goff et al. 2009; Lamien et al. 2011).

### Susceptible hosts

Sheep and goats are vulnerable to SPP and GTP. Many of the strains studied cause severe disease in either sheep or goats, but some have equivalent pathogenic effects in both species (FAO 2017b). By contrast, LSDV affects cattle and domestic Asian water buffaloes, but it does not naturally infect sheep and goats. However, some strains of LSDV can replicate in sheep and goats. Additionally, no epidemiological confirmation exists concerning the role of sheep and goats as reservoirs for LSDV (Tuppurainen et al. 2018). In Ethiopia, GTPV and LSDV have been reported for CaPV outbreaks in small ruminants and cattle, respectively, whereas SPPV is absent (Gelaye et al. 2015). Additionally, according to Kenubih et al. (2021), GTPV has been accountable for the occurrence of CaPV diseases in small ruminants. Currently, no decisive confirmation exists that SPPV, GTPV (FAO 2017b), and LSDV can affect humans (OIE 2010; Haller et al. 2014).

### Epidemiology

#### Geographic distribution

**Fig. 1:**
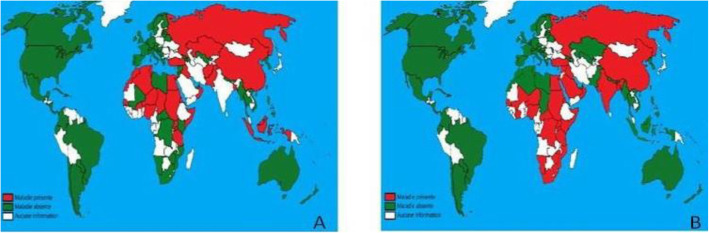
**A** Sheep pox geographic distribution. **B** Lumpy skin disease geographic distribution. Source: (Hamdi et al. 2021). The red color indicates the presence of CaPV

CaPV infections in small ruminants have a global distribution and are wider than LSD (Fig. 1A). The distributions of CaPV in small ruminants are relatively stable. Numerous studies and reports suggest that SPP and GTP viruses are highly distributed in northern and central Africa, the Middle East, Europe and Asia (Tuppurainen et al. 2017). However, LSDV has significantly increased in Asia, including Russia, China, India, Thailand, Malaysia, Sri-Lanka, Bangladesh and Vietnam (Fig. 1B) (FAO 2017a; Hamdi et al. 2021), and frequent outbreaks of LSD in Greece were reported in the past, with occasional outbreaks of LSD in Bulgaria due to outbreaks that occurred in Turkey (OIE 2008).

Sporadic outbreaks of SPP and GTP have been reported in Southern Europe (Rao and Bandyopadhyay 2000). During 2013, the most recent disease outbreaks were recorded in Bulgaria and Greece, Israel in 2014, and Russia and Mongolia in 2015 (Tuppurainen et al. 2017). In Ethiopia, very limited investigations have been conducted on sheep and goat pox virus in selected parts of the country (Gari et al. 2015). However, the diseases have a widespread distribution in all regional states of the country and affect the production and productivity of the subsector in the country. In the Gondar veterinary clinic, the prevalence was 40% in sheep and 8.12% in goats (Molla et al. 2017a); in the Gamo Gofa zone of the country, the prevalence was 31.96% in sheep and 35.28% in goats (Kebede et al. 2018). Recently, seroprevalence epidemiological studies have been performed on sheep and goat pox. However, the spatiotemporal clustering of SPP and GTP incidence rates has not been studied (Aregahagn et al. 2021).

#### Host-specificity

Maksyutov et al. (2015) reported that most CaPV strains are highly host-specific, with only some exceptions (Maksyutov et al. 2015). Thus, SPPV isolates cause disease mainly in sheep, and GTPV isolates cause disease primarily in goats (Bhanuprakash et al. 2010). However, both species of small ruminants may acquire the disease from a single strain of the virus. Some sheep strains cause mild disease in goats and severe disease in sheep, whereas virulent goat strains can infect sheep (Bhanuprakash et al. 2010; He et al. 2020). Thus, CaPVs are cross-reactive within the genus *Capripoxvirus* (Tuppurainen et al. 2014). SPPV and GTPV can cause cross-infection either naturally or experimentally (Davies 1982). However, no evidence has supported that LSDV can cause disease in small ruminants (USDA 2016).

#### Transmission

Transmission of CaPV is mainly considered by either direct contact with contaminated respiratory droplets or indirect contact through contaminated environments and vectors (Sprygin et al. 2019). However, these viruses are viable for extended periods in the environment. Thus, the movement of contaminated animals acts as the most important reason for the transmission of the agents (Rao and Bandyopadhyay 2000; Verma et al. 2011). Experimentally, sheep and goats have been infected with intradermal (Bowden et al. 2008) and intranasal inoculation of the respective viruses (Balinsky et al. 2007). According to Bhanuprakash et al. (2006), stable flies were confirmed to transmit SPP and GTP viruses mechanically.

#### Morbidity and mortality rates

SPPV and GTPV cause major economic disasters because of the relatively high morbidity and mortality of vulnerable animals. Thus, in endemic areas, the morbidity is between 75% and 100% and between 10% and 85% based on the virulence of the isolates (Bhanuprakash et al. 2006). Additionally, the mortality rate can reach up to 100% in stressed and susceptible animals (Domenech et al. 2006) but usually ranges from 5% to 10% in local breeds (Bhanuprakash et al. 2006).

Additionally, morbidity and case fatality rates of approximately 20% and 40% have been reported, respectively (Beard et al. 2010), while the mortality rate is not very high (up to 10%) because of LSDV (Calistri et al. 2018). In Ethiopia, SPPV and GTPV are highly distributed in all regions of the country and cause huge production losses and mortality (Yune and Abdela 2017). Species, stress, coexisting infection, breeds, age, host immunity, and virus isolates may all influence disease morbidity and mortality (Babiuk et al. 2009a; Tuppurainen et al. 2017). Native breeds are more resistant to CaPV than European breeds. Young animals are generally at greater risk than adults because of extensive interstitial viral pneumonia (Kitching et al. 1986).

### Economic Importance of sheep and goat pox

Certain infectious diseases, such as SPP and GTP, are the most important viral diseases of small ruminants that cause severe production losses in sheep and goats in the diseases’ respective endemic areas. The disease also limits international trade and causes other economic losses (Tuppurainen and Oura 2012; Hopker et al. 2019). Hence, the World Organization for Animal Health (OIE) has classified CaPV as a notifiable disease because of their rapid transboundary nature and extensive economic impact on the livestock industry (Tuppurainen and Oura 2012; Zeng et al. 2014; Hamdi et al. 2021). Similarly, these economically devastating viral diseases affect sheep and goats. Globally, the disease causes a serious risk to small ruminant production and food security and jeopardizes international trade (Tuppurainen et al. 2015).

Furthermore, CaPV can cause significant economic losses due to control costs and trade restrictions. In an endemic area, the economic losses of SPP and GTP have been reflected by reduced milk and mutton production, decreased weight, abortion, significant harm to wool and hides, and vulnerability to pneumonia and fly strike. The disease’s direct economic impacts are primarily due to higher morbidity rather than mortality in susceptible animals (Al-Salihi and Hassan 2015; Molla et al. 2017b). In India, the estimated morbidity and death rates of 63.5% and 49.5% were reported, respectively, for sheep and goat production (Bhanuprakash et al. 2011). However, a significant decrease in milk production (up to 30%), an increased death rate in experimentally infected animals (up to 95%), and a decrease in conception rate following the outbreak of the disease in Israel have been observed (Yeruham et al. 2007).

Depending on the current scenario in Ethiopia, small ruminant production is a basic source of income and food for the small farmholder community in the country, and it has a high potential for foreign exchange earnings. Additionally, small ruminant production is considered a savings sector for smallholder farmers in addition to a source of income and food because it eliminates threats to disadvantaged communities in the absence of crop production due to natural disasters. Furthermore, other socioeconomic and cultural functions are involved for small-holder households. During 2010/11, export values of 63 million USD from meat and 148 million USD from live animals were acquired from small ruminants (Haile et al. 2018).

Similarly, the present exploitation of hides and skins is expected to be 75% for goat skin and 97% for sheep skin, with the estimated annual off-take rates of sheep and goats of 33% and 35%, respectively. The country supplies many finished and semifinished small ruminant hides and skins to the global market and accounts for approximately 12-16% of the total value of its exports (Yacob et al. 2008; Zemene and Mekonnen 2012). Several factors, however, such as sheep and goat CaPV, have hampered the sector’s development in the country (Yune and Abdela 2017; Kenubih et al. 2021).

Furthermore, a lack of detailed information or insufficient information has contributed substantially to the prevalence of SPP and GTP in Ethiopia. Consequently, 893 outbreaks were reported in 2007/08. Of these eruptions, 57638 small ruminants became sick, among which 6401 (11.1%) died (Entity 2016). Additionally, from 2010 to 2014, 366 outbreaks were reported. Of these eruptions, 12822 were sick, and 1480 died of sheep pox; from 182 outbreaks, 10066 were infected, and 997 deaths were reported from goat pox in the Amhara Regional State (Fentie et al. 2017).

Furthermore, the annual economic losses due to mortality range from 12 to 14% for sheep and 11 to 13% for goats, as reported previously (MOA 2013). Hence, these diseases present major limitations to global trade and prevent the introduction of improved breeds of animals into endemic regions, being responsible for a significant economic impact on the animal industry in Ethiopia. These diseases are more severe in the lowland than in the midland and highland agro-ecologies of the country (Fentie et al. 2017).

## Diagnosis

### Clinical diagnosis

Clinically, it is difficult to distinguish SPP from GTP based on symptoms, lesions, and postmortem lesions (Bowden et al. 2008). Additionally, in the field, many clinical and pathological manifestations are recognized because of variations in the host response, virus species and virulence of the viral strains (Sumana et al. 2020). The incubation time differs from 4 to 21 days (Gitao et al. 2017); however, it is generally 21 days (OIE 2010). In general,CaPV infections have similar clinical manifestations (Rao and Bandyopadhyay 2000).

**Fig. 2:**
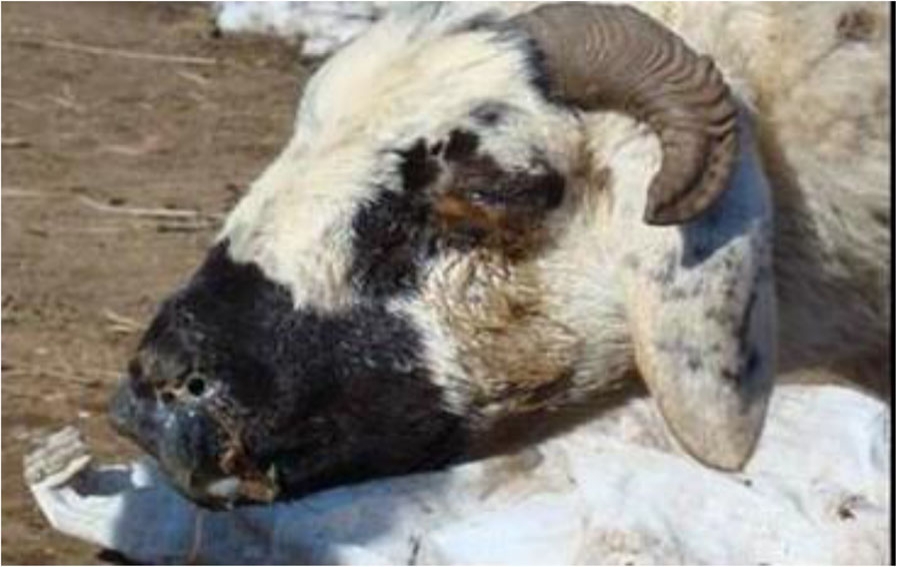
Thick discharges from the nose, nostrils and eyes. Source: (Mirzaie et al. 2015)

Initially, the lesions appear as papules and further progress to nodules, vesicles and pustules (raised lesions); finally, scab formations are detected on the skin (Babiuk et al. 2008; Bhanuprakash et al. 2006). Most affected animals become weak and lose appetite, have a high fever (40-42 °C), and find it difficult to breathe because of the presence of blisters inside their respiratory tract and lungs (Bowden et al. 2008). Skin lesions are visible on the entire body of infected animals but can be easily seen on hairless areas. Lesions (mouth, nose and eyelids), nasal discharge, and extreme salivation (Fig. 2) occur. Mucous membranes become necrotized and ulcerative. The presence of nodules in the intestine leads to diarrhea (Rao and Bandyopadhyay 2000; Haller et al. 2014).

### Differential diagnosis

SPP and GTP may be confused with contagious ecthyma, dermatophilosis, sheep scab, urticaria, parasitic pneumonia, and mange. However, in severe cases, clinical signs are highly characteristic. Additionally, a simultaneous infection with orf and peste des petits ruminants can cause the clinical signs of SPP or GTP with skin lesions occurring from orf and lung lesions and nasal discharge from PPR (OIE 2008).

### Pathology and post-mortem diagnosis

#### Gross lesions

**Fig. 3:**
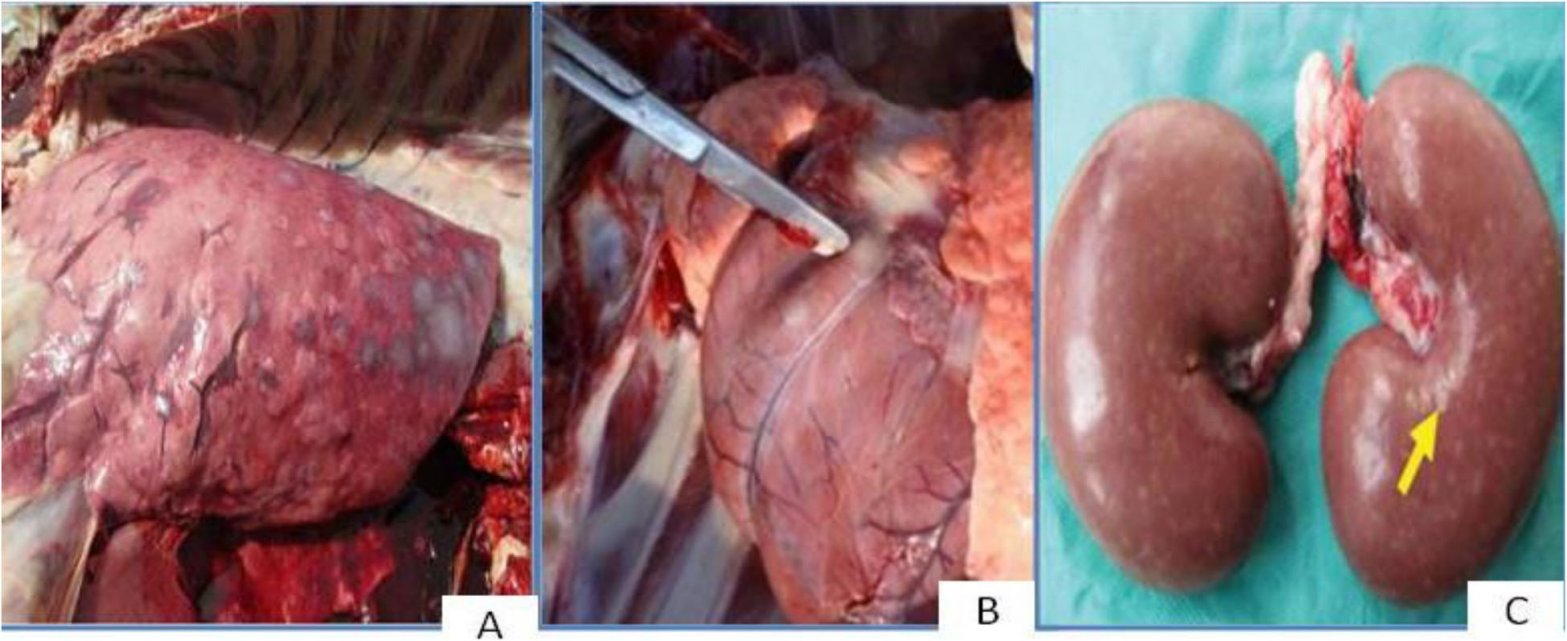
Pox lesions in **A** the lung, **B** heart muscles and **C** kidney. Source (Courtesy: photograph Colin Scrivener)

At postmortem examination, Pox lesions were distributed throughout the lung, kidney, heart (Fig. 3), and digestive and respiratory tracts.

**Fig. 4:**
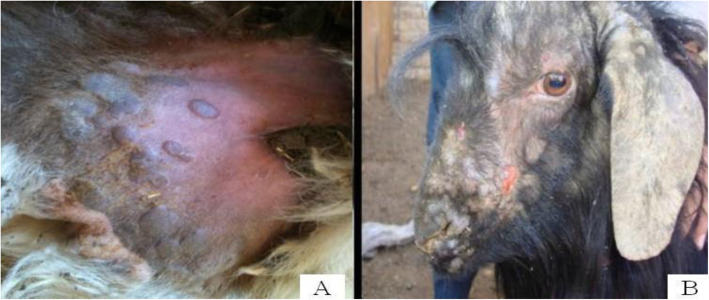
Popular lesions under the tail (**A**). Source (Zangana and Abdullah 2013). Pox lesions on the face and ears (**B**). Source (Mirzaie et al. 2015)

Popular lesions were also accumulated over the hairless areas of infected animals, such as the tail (Fig. 4A), face and ear (Fig. 4B). Additionally, lesions were detected throughout the entire body, including on the tail, face, lips, nose, muzzle, eyelids, ear, flank, abdomen, vagina, udder and all limbs (Gitao et al. 2017).

#### Histopathological lesions

In a histopathological study, the affected parts of the skin layers (epidermal and dermal changes) were characterized by hyperkeratosis, acanthosis, hyperkeratinization, edema, degenerative changes of sebaceous glands and hair follicle-affected parts (Pham et al. 2020). Pham et al. (2020) and Afshar et al. (1986) also reported pulmonary lesions and proliferative alveolitis with infrequent cytoplasmic inclusions in alveolar cells and macrophages.

Furthermore, sheep and goats were inoculated with SPPV and GTPV in their respective homologous hosts for detailed histopathological examinations. A monoclonal antibody generated from SPPV was utilized for immunohistochemical detection of viral antigen. Lesions and antigens were reported constantly in the skin, lung and lymph nodes. In general, recent histological findings revealed similarities between monkey pox and smallpox. Thus, CaPV infection in small ruminants may be a useful model to research strategies for poxvirus-specific virus vaccine conceptions and therapeutics (Embury-Hyatt et al. 2012).

Laboratory diagnosis is necessary to confirm the disease, and the following methods are used. It includes serological tests such as serum neutralization tests (more sensitive and specific gold standard) (Wolff et al. 2020), indirect fluorescent antibody test (IFAT), enzyme-linked immunosorbent assay (ELISA), and agar gel immunodiffusion assay (AGID) (Milovanović et al. 2019). Virus neutralization tests (VNTs) are the gold standard technique (Haegeman et al. 2016). However, VNT may not detect low levels of antibodies from vaccinated animals (Babiuk et al. 2009b; Gari et al. 2008).

Viral isolation is required to validate the infectivity of the agent. Thus, primary cell lines can be used to isolate SPPV from GTPV (Babiuk et al. 2007). Additionally, PCR is a simple and convenient technique to detect CaPV genomes in tissue samples (Lamien et al. 2011), but it is impossible to discriminate between GTPV and SPPV (Rao and Bandyopadhyay 2000). However, sequencing of the *P32* (Zeng et al. 2014) and*GpCR* genes was used to distinguish these viruses (Yan et al. 2012; Sumana et al. 2020). Additionally, a specific and sensitive duplex PCR technique was applied to the differential analysis of GTPV and SPPV, particularly in resource-limited endemic countries (Zhao et al. 2017). Likewise, real-time PCR containing a snapback primer and a DNA intercalating dye (Gelaye et al. 2013) and a real-time high-resolution melting PCR assay have been developed (Pestova et al. 2018).

Electron microscopy can distinguish CaPV morphology from *parapoxvirus* morphology (Balinsky et al. 2008). *CaPV*  CaPV can be easily identified by laboratory examinations of other poxviruses that impart similar clinical manifestations in animals. Hence, CaPV strains can be obviously distinguished by genetic sequencing (Lahens et al. 2017; Biswas et al., 2020b). Thus, full genetic sequence examination have revealed that these viruses are distinct from each other and are classified into three distinct groups. However, GTPV and LSDV are more similar than SPPV to LSDV (Le Goff et al. 2009; Lamien et al. 2011). Thus, sequencing of *GPCR* and *P32* has been applied to identify and differentiate LSDV from SPPV and GTPV (Mafirakureva et al. 2017; Salnikov et al. 2018). However, the *P32* gene is a better marker than LSDV termini to distinguish different CaPV strains (Sameea Yousefi et al. 2018).

## Treatment, control and prevention

No specific treatment is available for a viral disease, but antibiotics are given for secondary bacterial infection, and good nursing care is recommended to reduce morbidity and other complications (Hajer et al. 1988). Additionally, better consideration of disease occurrence and its distribution would lead to improved control measures (Fentie et al. 2017; Limon et al. 2020). Effective control and eradication of CaPV of small ruminants in previously CaPV-free countries could be practiced by slaughtering all contaminated and in-contact animals (Tuppurainen and Galon 2016), given that the application of attenuated vaccines in nonendemic areas may not be advantageous (Bowden et al. 2008). However, in many endemic countries, live attenuated vaccines provide longer-term protective immunity than inactivated vaccines (Bhanuprakash et al. 2012; Welfare, E. P. on A. H. and 2014).

Furthermore, live attenuated Capripox vaccines are safe and effective to combat these diseases (Tuppurainen et al. 2017), while inactivated vaccines do not offer prolonged immunity and require two doses (Boumart et al. 2016). Although different studies have shown that, even in the face of adversity (e.g., insufficient protection and short-lived), recent findings strongly support that inactivated Capripox vaccines are safe and efficacious against CaPV (Wolff et al. 2021; Es-sadeqy et al. 2021). In CaPV infection, both antibody and cellular immunity provide lifelong immunity (Bhanuprakash et al. 2006). However, cell-mediated immunity elicits long-term protection (Carn 1993). The close antigenic relationship (which shares 98% sequence similarity) among SPP, GTP and LSD theoretically allows for a single vaccine to protect against all these diseases (Brenner et al. 2009).

In Ethiopia, annual mass vaccination with the Kenyan sheep and goat pox (KSGP) O-180 virus strains has been demonstrated to be a safe, effective and affordable method to control small ruminant pox virus (Fentie et al. 2017). However, the absence of adequate infrastructure could hinder the implementation of sufficient herd immunity (Mirzaie et al. 2015; Barua et al. 2017). In Ethiopia, approximately 22 different types of vaccines have been produced for domestic use and the export market. Among these, the live attenuated (KSGPV) O-180 vaccine strain has been manufactured using continuous cell lines (Vero cells) and is a lyophilized vaccine with a stabilizer that is stored at − 20 °C for 2 years. Although some cattle breeders question whether the KSGP O-180 vaccine provides adequate protection, the KSGP vaccine strain is used currently to control LSD in cattle. However, this strain in Ethiopia is safe and effective in controlling sheep and goat pox if and only if storage, transportation, handling of the vaccines, and sufficient vaccination coverage are properly maintained.

Mutations of ankyrin (ANK) and kelch-like proteins of some of the CaPV vaccine strains have recently been reported. The *ANK* gene of CaPV makes it a potential candidate to develop a CaPV vaccine. Thus, many live vaccines of CaPV present a familiar attribute that *ANK* genes have mutated (Biswas et al. 2020). By contrast, vaccine failure may be associated with insufficient vaccination coverage and the production of low-performance local vaccines (Gelaye et al. 2015), and attenuated vaccine isolates are clinically secure to control disease in sheep and goats but not in cattle (Ayelet et al. 2013). According to Liu et al. (2019), the characteristics and attenuation properties of the virus strain must be determined while selecting vaccine strains to immunize cattle, sheep and goats. Additionally, *CaPV* vaccines require evaluation in all animal species to determine the their efficiency (Teffera and Babiuk 2019).

## Conclusions

Sheep pox and goat pox are distributed worldwide, including in Ethiopia. Transmission is thought to facilitated by either direct or indirect contact with respiratory droplets of acutely contaminated animals. SPP and GTP have huge economic consequences for livestock owners in endemic areas and are the main limitations on the international market for live animals and their products. Economic losses are also reflected by decreased meat and milk production, abortion, low quality of wool and skin, and the international trade ban. Many live-attenuated vaccines of CaPV were mutated and ultimately failed to protect animals from infection. Conversely, vaccine failure could be associated with insufficient coverage and vaccine quality. In Ethiopia, the National Veterinary Institute has been working extensively to improve the current KSGP vaccines against these infections. Livestock owners understand the benefits of vaccines and have a strong desire to vaccinate their flocks. However, most livestock owners, particularly in rural areas, have a low level of education, and significant gaps exist regarding the use and management of these vaccines. Additionally, the existing KSGP-based attenuated vaccine whole genomes should be sequenced for the development of new, safe and more efficacious vaccine candidates for small ruminants and cattle to maintain better control of CaPV in the country. Furthermore, awareness-raising campaigns for farmers to promote vaccine management and handling should be considered.

## Data Availability

Not applicable.

## Abbreviations

AGID: Agar gel immunodiffusion assay
ANK: Ankyrin
CaPV: Capripoxviruses
ELISA: Enzyme-linked immunosorbent assay
GpCR: G-protein coupled receptor
GTP: Goat pox
GTPV: Goat pox virus
IFAT: Indirect fluorescent antibody test
KSGP: Kenyan sheep and goat pox
LSDV: Lumpy skin disease virus
NVI: National Veterinary Institute
OIE: World Organization for Animal Health
PCR: Polymerase chain reaction
SPP: Sheep pox
SPPV: Sheep pox virus
USD: United States dollar
VNT: Virus neutralization tests
